# Pneumococcal surface adhesion A protein (PsaA) interacts with human Annexin A2 on airway epithelial cells

**DOI:** 10.1080/21505594.2021.1947176

**Published:** 2021-07-08

**Authors:** Yoonsung Hu, Nogi Park, Keun Seok Seo, Joo Youn Park, Radha P. Somarathne, Alicia K. Olivier, Nicholas C. Fitzkee, Justin A. Thornton

**Affiliations:** a Department of Biological Sciences, Mississippi State University, Mississippi State, USA; b Department of Basic Sciences, College of Veterinary Medicine, Mississippi State University, Mississippi State, USA; c Department of Chemistry, Mississippi State University, Mississippi State, USA; d Department of Population and Pathobiology, College of Veterinary Medicine, Mississippi State University, MS, USA

**Keywords:** *Streptococcus pneumoniae*, surface protein adhesin A (PsaA), Annexin A2 (Anxa2), host cellular receptor, protein vaccine, adhesion, colonization

## Abstract

*Streptococcus pneumoniae* (pneumococcus) is a normal colonizer of the human nasopharynx capable of causing serious invasive disease. Since colonization of the nasopharynx is a prerequisite for progression to invasive diseases, the development of future protein-based vaccines requires an understanding of the intimate interaction of bacterial adhesins with host receptors. In this study, we identified that pneumococcal surface adhesin A (PsaA), a highly conserved pneumococcal protein known to play an important role in colonization of pneumococcus, can interact with Annexin A2 (ANXA2) on Detroit 562 nasopharyngeal epithelial cells. Lentiviral expression of ANXA2 in HEK 293 T/17 cells, which normally express minimal ANXA2, significantly increased pneumococcal adhesion. Blocking of ANXA2 with recombinant PsaA negatively impacted pneumococcal adherence to ANXA2-transduced HEK cells. These results suggest that ANXA2 is an important host cellular receptor for pneumococcal colonization.

## Introduction

*Streptococcus pneumoniae* (pneumococcus) is gram-positive bacterium that commonly colonizes the nasopharynx of humans and is capable of causing invasive pneumococcal disease (IPD), including pneumonia, bacteremia, and meningitis [1]. The currently available vaccines in the US are the 13-valent pneumococcal conjugate vaccine (PCV13) and the pneumococcal polysaccharide vaccine (PPSV23) [2]. These vaccines have proven their efficacy against most IPD-causing vaccine serotypes [3]. However, these vaccines are less effective at preventing colonization and infection of non-vaccine serotype pneumococcal strains; consequently, diseases caused by these non-vaccine strains are increasing [4]. Since the introduction of the current vaccines, colonization of nonvaccine serotypes has increased and these nonvaccine serotypes are highly resistant to more than 2 different classes of antibiotics [5,6]. Furthermore, pneumococci are one of the several naturally competent bacteria that are capable of taking up free DNA from other pneumococci *in vivo* [7,8]. This has resulted in a serotype replacement in the face of vaccine pressure [9,10]. Therefore, the development of protein-based vaccines targeting protein antigens that are commonly present in pneumococci may resolve the issue of current serotype-dependent vaccines.

The human nasopharynx is the major colonization site for pneumococcus and colonization is generally prerequisite for invasive disease [11]. During colonization, pneumococcus expresses various surface proteins and enzymes that interact with the host epithelial cells and facilitate attachment to the upper respiratory tract of humans [12]. Thus, pneumococcal surface proteins and enzymes are ideal vaccine targets to interfere with the first step of pneumococcal pathogenesis [13]. So far, more than 30 of pneumococcal surface proteins have been characterized in their role in colonization and subsequence form of infections [12]. Among them, the pneumococcal surface protein adhesin A (PsaA) is a lipoprotein component of an ABC transporter for manganese (Mn) which has been shown to contribute to colonization in humans [14]. Previous studies demonstrated that a PsaA-negative mutant displayed lower adherence to human nasopharyngeal epithelial cells (Detroit 562) and human lung alveolar epithelial cells (A549) compared to wildtype [15,16]. Since attachment of pneumococcus to the epithelium is essential for the development of pneumococcal disease, identifying the host cellular receptors of PsaA and other adhesins is critical for the development of vaccines effective at preventing colonization. Furthermore, PsaA is highly conserved in most pneumococcal serotypes and is immunogenic in all age groups which makes it a potential vaccine candidate [17]. A previous study identified E-cadherin as a potential receptor for PsaA on human nasopharyngeal cells [18]. We hypothesized that PsaA could interact with additional epithelial receptors and here describe the interaction of PsaA with human Annexin A2 (ANXA2).

## Materials and methods

### Streptococcus pneumoniae *chromosomal DNA isolation*

*Streptococcus pneumoniae* strain TIGR4 was grown on tryptic soy agar supplemented with 5% of sheep blood for overnight at 37°C in 5% CO_2_ then inoculated into 20 ml of C + Y media and grown to a mid-logarithmic phase (O.D._600_nm of 0.6) in a 37°C water bath [19]. The cells were collected by centrifugation at 6000 rpm for 10 min and suspended in 500 µl of TES buffer containing 10 mM Tris-HCl (pH 7.6), 1 mM EDTA, and 0.5% w/v SDS. The suspension was centrifuged at 6000 rpm for 1 min and resuspended in 500 µl of TES buffer prior to the following steps: addition of 50 µl of 1% Triton X-100 and incubation at 37°C for 15 min, addition of 50 µl RNase (10 mg/ml) and incubation at 37°C for 15 min, addition of 20 µl Proteinase K and incubation at 37°C for at least 2 hr. An equal amount of phenol-chloroform-isoamyl alcohol (25:24:1) was added and vortexed vigorously. The suspension was centrifuged at 13,000 rpm for 10 min. The aqueous layer was transferred to a new tube and equal volume of chloroform was added. The suspension was vortexed vigorously and centrifuged at 13,000 rpm for 10 min. Again, the aqueous layer was collected and two volumes of 100% ethanol was added. The ethanol suspension was centrifuged at 13,000 rpm for 10 min and supernatant was discarded. The pellet was washed with 70% of ethanol and centrifuged at 13,000 rpm for 15 min. The supernatant was discarded, and excess ethanol was air dried. The air-dried pellet was resuspended in 50 µl of TE buffer (10 mM Tris-base, 1 mM EDTA, pH 8.0).

## Gene cloning and expression of recombinant PsaA

The *psaA* gene was amplified from TIGR4 DNA using Ex-taq DNA polymerase (Takara, Cat. RR001A) following manufacturer’s instructions. Both pOS1 customized vector for protein expression and purification and PCR products were digested with BamHI and XhoI restriction enzymes (New England Biolabs). Following restriction enzyme digestion, the *psaA* gene was ligated with pOS1 customized staphylococcal expression vectors that can secrete the protein into the bacterial culture media. The ligation product was transformed into a chemically competent *E. coli* DH5α cells [20]. The purified plasmid was extracted from *E. coli* and electroporated into *S. aureus* strain RN4220 as previously described [21]. The positive *S. aureus* transformant was confirmed by PCR and grown overnight in 1 ml of YCP media (yeast extract, casamino acids, pyruvate, salts) supplemented with 10 µg/ml of chloramphenicol at 37°C [22]. Following the incubation, 100 µl of culture was transferred to 50 ml of fresh YCP media supplemented with 10 µg/ml of chloramphenicol and incubated at 37°C overnight and sterile filtered the following day. The filtered culture supernatant was added to nickel resin to purify the 6x histidine tagged PsaA by nickel chromatography. The protein purification was confirmed by SDS-PAGE (Figure S1).

## Biotin labeling of PsaA

The purified PsaA protein was biotinylated using EZ-Link™ NHS-LC-Biotin (ThermoFisher Scientific #21,327) according to the manufacturer’s instruction. The excess non-reacted biotin reagent was removed by PD-10 desalting column (GE Healthcare #17,085,101) and biotinylation was confirmed by western blot using HRP conjugated streptavidin.

## Extraction of nasopharynx epithelial cell lysate proteins and two-dimensional electrophoresis

Detroit 562 human nasopharyngeal epithelial cells were grown in EMEM medium (ATCC, 30–2003) containing 10% fetal bovine serum, 2 mM L-glutamine, and penicillin/streptomycin in T-75 culture flasks, collected by scraping, washed with PBS and transferred to microcentrifuge tubes. The cell pellets were resuspended in 400 µl of TE buffer (10 mM Tris, 1 mM EDTA, pH 8.0) and 650 µl of Phenol:Chloroform:Isoamyl alcohol (25:24:1) and vortexed vigorously. The suspension was centrifuged at 13,000 rpm for 15 min and both aqueous and organic phases were discarded. The interphase was suspended in 1 ml of chloroform, vortexed vigorously, and centrifuged at 13,000 rpm for 5 min. The chloroform layer was removed and the pellet was air dried. The pellet was resuspended in a rehydration buffer (7 M urea, 2 M thiourea, 0.5% triton X-100, 2 mM TBP). The total amount of protein in the samples was quantified using 2D Quant kit (GE Healthcare) and 100 µg of proteins were separated by 12.5% SDS-PAGE or 2 DE using 3–11 non-linear pH gradient 7 cm IPG strip (GE Healthcare, #GE17-6003-73). Isoelectric focusing was performed using a Multiphor II electrophoresis system (GE Healthcare) in three running phases (phase I, 200 V/0.01 h; phase II, 3500 V/1.5h; phase II, 3500 V/1.05 h). The strip was equilibrated in 1% dithiothreitol, followed by 5% iodoacetamide for 10 min in each. The second dimension SDS-PAGE was performed using 12.5% polyacrylamide gels in duplicate. One gel was stained with colloidal Coomassie (Pierce) and the other gel was transferred to a PVDF membrane for far western blot analysis.

## Far-western blot and mass spectrometry

The PVDF membrane was incubated in AC buffer (100 mM NaCl, 20 mM Tris pH7.6, 0.5 mM EDTA, 10% glycerol, 0.1% Tween 20, 2% skim milk) with a step-wise reduction of the guanidine-HCl concentration from 6 M, 3 M, 1 M, 0.1 M, and 0. The PVDF membrane was incubated with 100 µg of biotinylated PsaA in PBST buffer (4 mM KH_2_PO_4_, 16 mM Na_2_HPO_4_, 115 mM NaCl, pH 7.4) containing 2% skim milk overnight at 4 C. After washing with PBST buffer, PVDF membranes were incubated with streptavidin conjugated with horseradish peroxidase (HRP) in PBST buffer with 2% skim milk. Binding of biotinylated PsaA to target protein was detected by enhanced chemiluminescence (ECL) reagent (ThermoFisher Scientific) and visualized by x-ray film exposure.

To identify epithelial proteins specifically interacting with PsaA, the protein spots in the Coomassie stained gel were aligned to the PVDF membrane. Corresponding spots from Coomassie stained gel were excised and digested with trypsin (Worthington). The peptide mass spectra were generated using MALDI-TOF/TOF (ABI4700) and protein identification was performed using ABI GPS Explorer software V3.5. Deduced peptide sequence was analyzed using the Mascot search engine using the SWISS-PROT database. This work was done in our Center of Biomedical Research Excellence “Omics” Core, which is a component of the Institute for Genomics, Biotechnology, and Biocomputing.

## ANXA2 recombinant protein

The full codon sequence of ANXA2 isoform 2 gene (NM_001002858.3) was ligated with pET 21b vector (Novagen, #69,741) following the manufacturer’s instructions. The ligation product was transformed into chemically competent *E. coli* DH5α cells and finally, in *E. coli* protein expression strain BL21 (DE3) (Novagen, #69,450). The transformant was induced with 1 mM of IPTG at 37°C for overnight. The IPTG induced bacterial cells were lysed with B-PER (ThermoFisher Scientific #78,243) following manufacturer’s instructions and soluble proteins were separated from the insoluble proteins by centrifugation at 10,000 × g for 10 min. The soluble proteins were added to nickel resin to purify the 6x histidine tagged ANXA2 by nickel chromatography and purified ANXA2 was confirmed by western blot. The purity of the protein is demonstrated in Figure S1.

## Mouse tissue lysates western blot

Murine tissues of a C57BL/6 mouse were excised from nasopharynx, bronchi, lungs, and heart. Tissues were homogenized by bead beating and lysed with modified hunter’s buffer (10 mM HEPES, 150 mM NaCl, 1.5 mM MgCl2, 1 mM EDTA, 10 mM Na-pyrophosphate, 10 mM NaF, 0.1 mM Na3VO4, 1% deoxycholic acid, 1% Triton X 100, 0.1% SDS). Following centrifugation of pellet debris, proteins from lysates were quantitated by BCA assay (ThermoFisher Scientific #23,227) and 25 μg from each tissue were subjected to SDS-PAGE on 12% polyacrylamide gel and transferred to PVDF membrane. The expression of ANXA2 was determined by western blotting probed with ANXA2 antibody (Santa Cruz #SC-28,385) followed by goat anti-mouse IgG HRP-conjugated secondary antibody (Bio-Rad #172-1011).

## ANXA2 immunohistochemical (IHC) staining

Murine tissues were excised along the respiratory tract (nasopharynx, bronchi, lungs), and heart. Tissues were fixed in fixed in formalin, embedded in paraffin, sectioned at 5 μm. The unstained slides were deparaffinized and pretreated with DIVA Decloaker (Biocare) in a Decloaking Chamber (Biocare) for 15 min at 110°C. Endogenous peroxidase activity was blocked with 3% hydrogen peroxide for 5 minutes. The 1:100 diluted ANXA2 primary antibody (Santa Cruz, sc-9061) was applied to the slides for 30 min. A negative control without primary antibody was included. Detection was performed using Rabbit-on-Canine HRP polymer (Biocare) according to insert directions. The tissues were developed with DAB chromogen substrate (Biocare) for 5 min. The slides were counterstained with hematoxylin (Biocare) for 5 min. The slides were washed, dehydrated, and coverslipped.

## Lentiviral expression of ANXA2

HEK293T cells were obtained from ATCC and cultured in a DMEM medium (ATCC, 30–2002) containing 10% fetal bovine serum, 2 mM L-glutamine, and penicillin/streptomycin (ThermoFisher Scientific). The lentiviral expression plasmid pLenti-C-Myc-DDK harboring the human annexin A2 (NM_001002858, pLenti-ANXA2) was obtained from Origene (RC215009). To generate lentiviruses, HEK293T/17 cells were transfected with pLenti-ANXA2 in combinations with the packaging plasmid, psPAX2 (Addgene) and the envelop plasmid pMD2.G (Addgene) using TransIT_293 transfection reagent (Mirus). After 24, 48, 72, 96 hours incubation, the culture supernatant containing lentiviruses was harvested and filtered through a 0.45-μm filter. Fresh HEK 293 T/17 cells were transduced with lentivirus for 12 hr and then cultured in a 48-well plate overnight. The following day, cells were transferred to 12-well plates and cultured overnight. On day 3, cells expressing ANXA2 were selected by culturing DMEM media containing 5 µg/ml of puromycin. For control, HEK 293 T/17 mock cells were made using empty transfer plasmid following same method as above. To confirm the expression of ANXA2, four different cell lines (Detroit 562, A549, HEK 293 T/17 Mock, and ANXA2 transduced HEK 293 T/17) were lysed with RIPA buffer (ThermoFisher Scientific, #89,901) following manufacturer’s instructions and were examined by western blotting probed with ANXA2 antibody (Santa Cruz #SC-28,385) followed by goat anti-mouse IgG HRP-conjugated secondary antibody (Bio-Rad #172-1011).

The full codon sequence of ANXA2 isoform 2 gene (NM_001002858.3) possessing signal sequence and a 6x histidine tag was ligated with pLenti6/V5 vector (Invitrogen #K495510). The ligation product was transformed into chemically competent *E. coli* Stbl3 cells (Invitrogen #C737303) by a general heat shock method. *E. coli* transformants were selected by ampicillin resistance and confirmed by PCR. The extracted plasmid from an *E. coli* transformant was used to generate the lentivirus in combination with the packaging plasmid, psPAX2 (Addgene), and the envelop plasmid pMD2.G (Addgene) using TransIT_293 transfection reagent (Mirus) following the same method as above. ANXA2 transduced HEK293 T/17 cells were selected by culturing DMEM media containing 5 µg/ml of blasticidin. The transduced cells were grown in DMEM medium containing 10% fetal bovine serum, 2 mM L-glutamine, and penicillin/streptomycin in T-75 culture flasks until 50% confluency. The medium was replaced with FreeStyle 293 expression medium (ThermoFisher Scientific, #12,338,018) and grown for another 7 days. The medium was collected and filtered. Secreted ANXA2 in the culture medium was purified by nickel chromatography and confirmed by western blotting using anti-ANXA2 antibody.

## Construction of PsaA knockout strain

A clean deletion PsaA knockout T4R strain was created using the pMBSacB plasmid [23]. Briefly, 1kb DNA segments from upstream and downstream of PsaA were amplified. The amplified upstream and downstream PCR products were cleaned and fused by PCR using outer primers. Both fused PCR product and pMBsacB plasmid, which is a suicide vector for site-specific mutations, were digested with BamHI and PstI restriction enzymes. The digested flanking construct and pMBsacB plasmid were ligated and transformed into chemically competent *E. coli* DH5α cells by a general heat shock method. The *E. coli* transformant was selected by erythromycin resistance selection and verified by PCR. The extracted plasmid from the *E. coli* transformant was transformed into *S. pneumoniae* strain T4R by standard *S. pneumoniae* transformation procedures [24]. Following selection and passage without erythromycin to allow for gene deletion recombination, pMBSacB-less PsaA deletion mutants were selected by 15% sucrose C + Y media [25]. The deletion of *psaA* gene was confirmed by PCR along with wild type as a control.

## Adhesion assay and blocking assay

HEK 293 T/17 mock transduced cells and ANXA2 transduced cells (≈ 10^6^ cells/well) were grown in penicillin-streptomycin free culture media for 2 days until confluent in 24-well plate. Cells were washed with serum-free media to remove serum. *S. pneumoniae* strain T4R (≈ 10^6^ CFU/well) was incubated with both cell types for 30 min at 37°C with 5% CO_2_ at a multiplicity of infection (MOI) of 1.0. Following incubation, eukaryotic cells were scraped, transferred to a microcentrifuge tube, and centrifuged at 800 rpm for 5 min. Nonadherent bacteria were removed by washing 3 times with DPBS and adherent bacteria were serially diluted and plated on 5% sheep blood agar supplemented with 5 µg/mL of chloramphenicol. The percentage of adherent *S. pneumoniae* was determined by comparison of CFU on blood agar after the final wash and initial number of bacteria. The assays were performed three times in triplicate.

For the competitive blocking assay, both HEK 293 T/17 mock and ANXA2 transduced cells were initially blocked with 200 µg of recombinant PsaA (rPsaA) in PBS for 1 hr at 37°C with 5% CO_2_ prior to incubation with bacteria. After the incubation with bacteria the number of adhered bacteria to human cells were determined by serial dilution and colony count.

## Detection of binding of ANXA2 to pneumococcus by flow cytometry

*S. pneumoniae* strains T4R (~1x10^5^ CFU) were resuspended in 1% BSA in PBS and divided into four different groups (~2.5 x 10^4^ CFU per each group): DAPI control, secondary antibody only control with recombinant ANXA2, both primary and secondary antibody control without recombinant ANXA2, and recombinant ANXA2 with both primary and secondary antibody. Each of four different groups were duplicated to test with two different concentrations of recombinant ANXA2 (20 µg and 100 µg). The recombinant ANXA2 group was incubated with recombinant ANXA2 on ice for 1 hr and washed with 1% BSA in PBS twice by centrifugation at 13,000 rpm for 5 min. Following the incubation with recombinant ANXA2, bacteria were incubated with 1:100 diluted ANXA2 antibody (Santa Cruz Biotechnology #SC-28,385) in 1% BSA in PBS for 1 hr at room temperature and washed with 1% BSA in PBS twice. The bacteria were incubated with 1:200 diluted FITC conjugated secondary antibody for 30 min at room temperature and washed with 1% BSA in PBS twice. Fluorescent intensity was quantitated using an Attune Acoustic Focusing Flow Cytometer (ThermoFisher Scientific) Bacterial cells were specifically gated based on duplicate samples stained with DAPI only.

## Characterization of PsaA and ANXA2 binding *in vitro*

The biophysical interactions of PsaA and ANXA2 were investigated using two different approaches: analytical size exclusion chromatography (SEC) and circular dichroism (CD) spectroscopy. For SEC, samples were prepared in PBS, and dithiothreitol (DTT) was added to a final concentration of 2 mM to reduce any oxidized disulfide dimers. The absence of dimers was later confirmed by running a non-reducing SDS-PAGE gel. The initial sample for SEC contained a 1:1 ratio of PsaA:recombinant ANXA2 expressed in *E. coli* and PsaA:ANXA2 expressed in HEK293 T/17 cells where each protein concentration was 0.18 mg/ml. The individual proteins and the protein mixtures were incubated for 1 hr at either 25°C or 37°C prior to analysis. Validation of PsaA – ANXA2 interactions was carried out using a Superdex 20,010/300 GL column connected to an AKTA Purifier 10 system at room temperature. Prior to the analysis, the column was equilibrated with two column volumes of PBS containing 2 mM DTT. A needle syringe was used to load 150 µL of the protein mixture into a 100 µL sample loop, and 100 μL was injected on to the column. The column was run at a flow rate of 0.5 mL/min according to the manufacturer’s specifications.

The CD measurements were carried out using a Jasco 1500 CD spectrometer at 25°C. The measurements were performed using a 1 mm pathlength quartz cuvette. Protein solutions were made containing 0.12 mg/ml of each protein, and mixtures contained a 1:1 ratio of PsaA and ANXA2 (*E. coli*) or ANXA2 (HEK293/T17), with each protein at 0.12 mg/ml concentration. The buffer for all solutions was 10 mM sodium phosphate (pH 6.5). The far-UV spectra were collected between 180 and 260 nm, with the scan rate set at 10 nm/minute at a bandwidth of 1 nm using 4 s as the integration time. All the spectra were smoothed using the Savitzky-Golay filter set to a window size of 17.

## PsaA and ANXA2 binding assay

Recombinant PsaA protein was covalently attached to AminoLink Plus beaded agarose resin (ThermoFisher Scientific, #20,394) following manufacturer’s instructions and modified protocol from a previous study [26]. For some samples, 5 mM of manganese sulfate was added to the PsaA-beads and incubated at 4°C for 30 minutes by end-over-end rocking to investigate if additional manganese affects binding. As a control, the BSA was covalently attached to resin and each bead was blocked with 50 mM NaCNBH_3_ for 30 minutes by end-over-end rocking after incubation with either PsaA or BSA. Control beads were blocked with 50 mM NaCNBH_3_ without the incubation with any proteins. The four different agarose beads: PsaA-bead, PsaA (Mn)-bead, BSA-bead, no protein-bead was incubated with ~50 µg of recombinant ANXA2 and BSA at 37°C for 1 hr, respectively. After incubation, beads were washed with PBS twice by centrifugation at 8000xg for 5 minutes. The remaining buffer was removed by 27 G x ½ needle attached to 1 ml syringe. The binding between bait and prey protein was disrupted by 40 µl of 2x Laemmli sample buffer (BioRad, #161-0737). The samples were heated at 95°C for 10 minutes and centrifuged at 13,000 rpm for 5 minutes. The supernatant was loaded onto an SDS-PAGE gel and binding of ANXA2 to PsaA was confirmed by western blotting using anti-ANXA2 antibody.

## Statistics

For adhesion assays, values from three or more experiments were compared by an unpaired, two-tailed Student’s t-test to test for statistical significance using GraphPad Prism version 9.0.0 for Windows (GraphPad Software, San Diego, California USA, www.graphpad.com). A P value less than 0.05 was considered significant.

## Results

### Identification of host cellular receptor for PsaA

**Figure 1.**
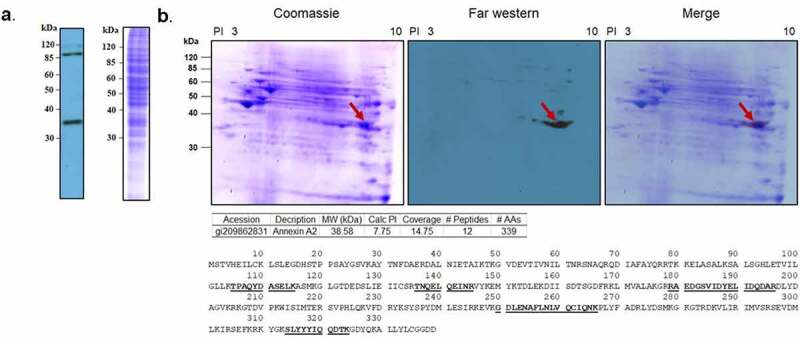
Far-western blotting of human nasopharynx cell and PsaA. (a) SDS-PAGE of whole cell lysate of human nasopharynx cell (Detroit 562) was probed with biotinylated PsaA. (b). Far- western blot analysis of whole cell lysate proteins of human Detroit 562 cell probed with biotinylated PsaA. A specific interaction between PsaA and whole proteins was detected by a streptavidin bead conjugated with HRP and visualized by x-ray film exposure. A protein spot from approximately 35 kDa (arrow) was excised from Coomassie stained gel and analyzed by electrospray ionization-tandem mass spectrometry for protein identification. Based on isoelectric point and molecular weight summarized in the table, human annexin A2 was identified as a putative receptor of PsaA. Amino acid sequences of human ANXA2 identified from mass spectrometry were underscored

To determine the interaction of PsaA with human pharyngeal epithelial cells, whole cell lysate proteins from Detroit 562 cells were separated by SDS-PAGE, followed by transferring to a PVDF membrane which was probed with biotinylated PsaA bait protein in a far-western blot assay. The specific interaction of PsaA with whole cell lysate proteins from Detroit 562 cell was visualized by HRP-conjugated streptavidin. A strong band was visualized at approximately 35 kDa (Figure 1a). Additional bands at approximately 90 kDa and fainter band at approximately 115 kDa were ruled out from further investigation since these were routinely visualized on control blots with only HRP-conjugated streptavidin. To identify potential receptors of PsaA, whole cell lysate proteins from Detroit 562 cells were further separated by 2DE, followed by far-westernblot analysis. A strong signal was observed at approximately 35 kDa. Mass spectrometry analysis identified 27 peptides. Based on isoelectric point and molecular weight, peptide coverage, and probability score, human annexin A2 (ANXA2) was identified as a putative receptor of PsaA (Figure 1b). For control, biotinylated HRP alone was used to probe a replicated membrane and no chemiluminescent signal was detected (data not shown).

## ANXA2 expression in the murine respiratory tract

**Figure 2.**
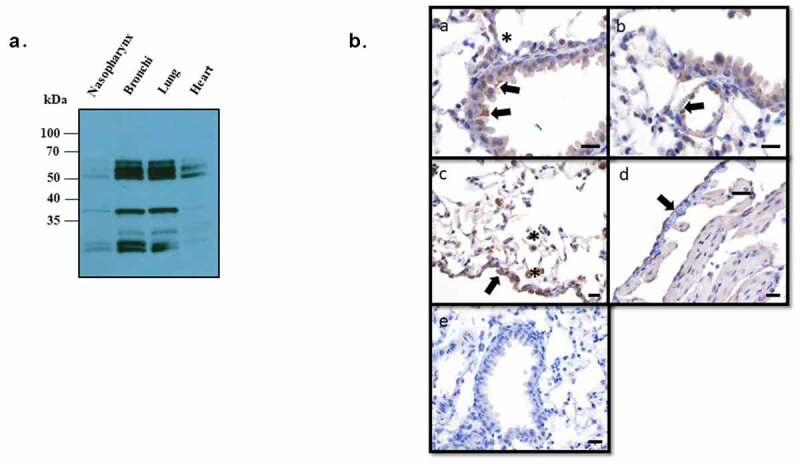
Expression of ANXA2 in murine respiratory tissues

Annexin A2 is expressed in a variety of tissues including respiratory epithelium. Results from Figure 2 demonstrates that Detroit 562 nasopharyngeal cells express ANXA2. To ensure this was not an artifact of immortalization and to evaluate relative expression throughout the respiratory tract, tissue from the nasopharynx, bronchi, lungs, and heart (as a control) were isolated from mice and subjected to western blot probing for ANXA2. A band of approximately 37 kDa corresponding to ANXA2 was highly expressed in the bronchi and lung and to a lesser degree in the nasopharynx, but not in heart tissue (Figure 2a).

(a) Tissues samples from the nasopharynx, bronchi, lung, and heart were collected from C57BL/6 J mice. Expression of ANXA2 in tissue homogenates were analyzed by western blot. (b) ANXA2 Immunohistochemical staining in murine lung and heart. (a) Within the lung staining was present along the apical surface of epithelial cells within bronchi and bronchioles (arrows) and rare type I pneumonocytes (asterisk). (b) Endothelial cells of small to medium sized vessels were diffusely positive (arrows). (c) Interstitial and alveolar macrophages (asterisks) had cytoplasmic staining and mesothelial cells along the visceral pleura of the lung were diffusely positive. (d) Within the heart staining was localized to vascular endothelial cells and visceral mesothelial cells (arrows), with no staining of cardiomyocytes. (e) Negative control. Bar = 50 µm.

Annexin A2 expression throughout the murine tissues were also detected by immunohistochemistry. The expression of ANXA2 was detected in multiple cell types and locations of lung. Within the bronchi and bronchioles there was multifocal staining of the apical membrane of the epithelial cells. Vascular endothelial cells of small to medium-sized vessels were diffusely positive but no staining was shown in cardiomyocytes. Within the interstitium and alveoli, macrophages have cytoplasmic staining. Diffusely, the mesothelial cells along the visceral pleura were diffusely positive (Figure 2b).

## ANXA2 transduced eukaryotic cells

**Figure 3.**
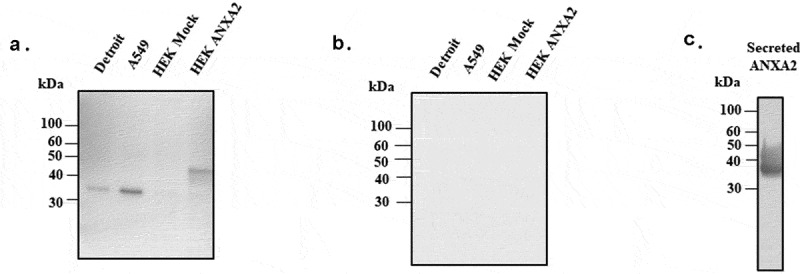
Western blotting probed with monoclonal ANXA2 antibody from mouse. (a) Four different cell lines (Detroit 562, A549, HEK 293 T/17 mock, and ANXA2 transduced HEK 293 T/17) were probed with ANXA2 antibody to detect the ANXA2 expression. Size difference could be due to lentiviral expressed plasmid containing Myc-DDK tag and multiple cloning regions. (b) Secondary only antibody control western blotting to confirm the nonspecific signal. (c) The purified ANXA2 by nickel chromatography from cell culture medium of ANXA2 transduced HEK293 T/17 cells

To verify the interaction of PsaA with ANXA2, we sought to express ANXA2 in naïve HEK293 T/17 cells using a lentiviral expression system. Expression of ANXA2 was assessed by western blotting using anti-ANXA2 antibody. As shown in Figure 3a, little to no expression was observed from HEK293 T/17 cells transduced with empty lentiviral expression vector, while HEK293 T/17 cells transduced with pLenti-ANXA2 expressed ANXA2 of greater molecular weight than that expressed in Detroit 562 cells (human nasopharyngeal) and A549 (human alveolar) epithelial cells. This is presumably due to addition of a MYC/DDK tag and multiple cloning site in pLenti-ANXA2. As a control, the PVDF membrane was probed with the secondary antibody only and no signal was detected (Figure 3b).

To further investigate the interaction of PsaA with ANXA2 *in vitro*, we expressed another ANXA2 in naïve HEK293 T/17 cells using a lentiviral expression system that can secrete ANXA2 out to culture media for purification. As shown in Figure 3c, this lentiviral system could express and secrete out the ANXA2 in cell culture medium.

## Effect of ANXA2 on colonization by pneumococcus

**Figure 4.**
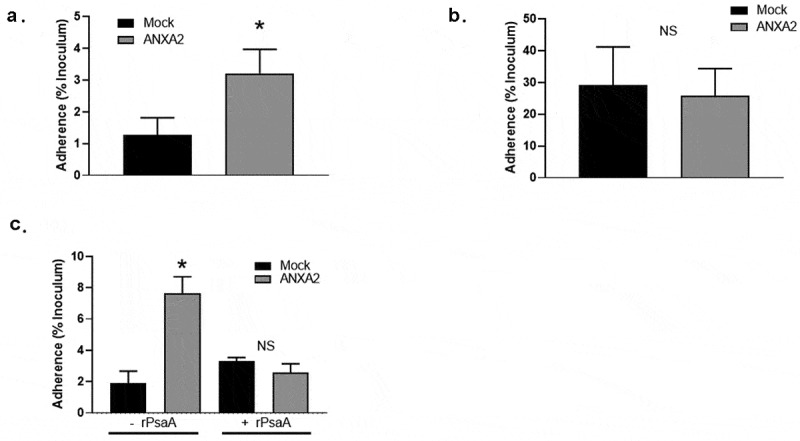
Binding of *S. pneumoniae* T4R to HEK 293 T/17 expressing ANXA2. HEK 293 T/17 expressing ANXA2 or Mock cells were incubated with *S. pneumoniae* T4R wild type (a) and at MOI of 1 for 30 min. After washing, the numbers of *S. pneumoniae* T4R bound to HEK 293 T/17 cells were determined by plate counts (b) T4R ΔPsaA at MOI of 1 for 30 min. After washing, the numbers of *S. pneumoniae* T4R bound to HEK 293 T/17 cells were determined by plate counts. (c) To determine the effect of blocking ANXA2 on binding of *S. pneumoniae* T4R to HEK 293 T/17 expressing ANXA2, HEK 293 T/17 expressing ANXA2 or Mock cells were incubated with recombinant PsaA (200 µg/ml) for 60 min. After washing, the numbers of *S. pneumoniae* T4R bound to HEK 293 T/17 cells were determined by plate counts. Experiments were performed three times in triplicate. Data shown are the mean ±SD. Asterisk indicates the statistical significance of student t-test at P < 0.05

Since PsaA is known to promote adherence of pneumococcus to human nasopharynx, we assessed the adherence of capsule null strain T4R to HEK293 T/17 cells transduced with ANXA2 and HEK293 T/17 mock transduced cells. T4R strain showed approximately 3.21 ± 0.76% adherence to HEK293 T/17 cells transduced with ANXA2, which was significantly higher than that of HEK293 T/17 mock transduced cells (1.23 ± 0.55) (Figure 4a). However, a ΔPsaA T4R isogenic mutant bound equally to mock and ANXA2 transduced cells (Figure 4b).

To confirm the specific interaction of PsaA with ANXA2 is responsible for the increased adherence of T4R to HEK293 T/17 cells transduced with ANXA2, we preincubated cells with recombinant PsaA, prior to incubation with *S. pneumoniae* T4R. Adherence of T4R to HEK 293 T/17 cells transduced with ANXA2 was significantly reduced by preincubation with PsaA, whereas adherence of T4R mock transduced cells was not (Figure 4c).

**Figure 5.**
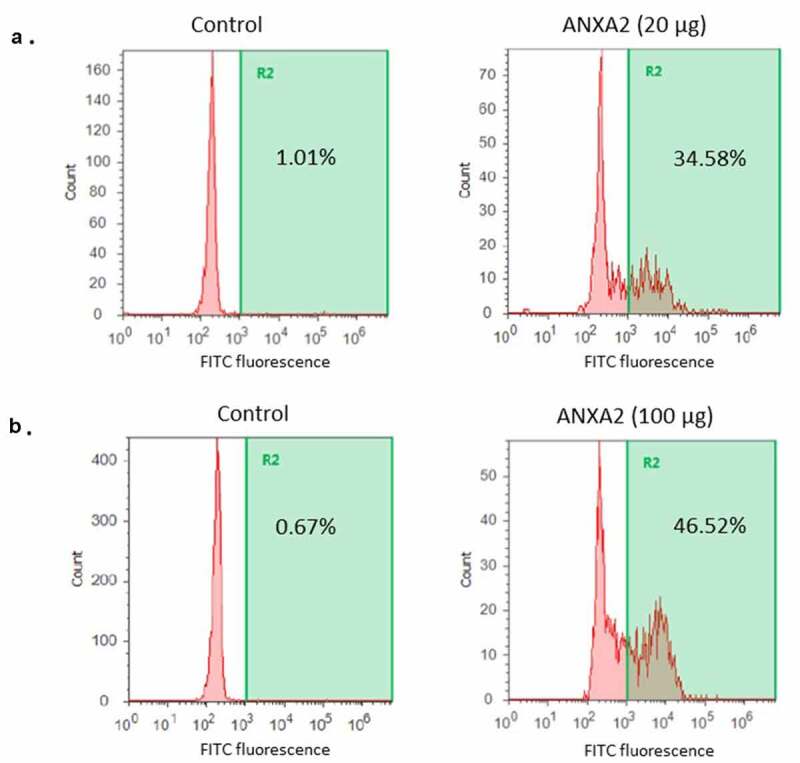
Binding of recombinant ANXA2 to *S. pneumoniae* T4R. A recombinant ANXA2 20 µg (a) or 100 µg (b) was incubated with *S. pneumoniae* T4R, followed by incubation with fluorescein isothiocyanate-conjugated anti-ANXA2 antibody. Binding of ANXA2 to *S. pneumoniae* T4R was assessed by flow cytometry. As a control, *S. pneumoniae* T4R was incubated with fluorescein isothiocyanate-conjugated anti-ANXA2 antibody. Percentages shown are percent of the total population positive for FITC as compared to secondary only antibody control with each different amount of recombinant ANXA2. Data are representative of at least three replicate experiments

To further confirm binding of ANXA2 with pneumococcus, strain T4R strain was incubated with 20 µg or 100 µg of recombinant ANXA2. The binding of ANXA2 to pneumococcus was quantitated by flow cytometry after incubation with ANXA2 antibody, followed by fluorescein conjugated secondary antibody. As shown in Figure 5, the binding of ANXA2 to pneumococcus increased in a dose dependent manner. Combined, these results demonstrate that PsaA binds to ANXA2 and this interaction can promote pneumococcal adherence to human nasopharyngeal cells.

## Biophysical measurements of PsaA:ANXA2 binding

**Figure 6.**
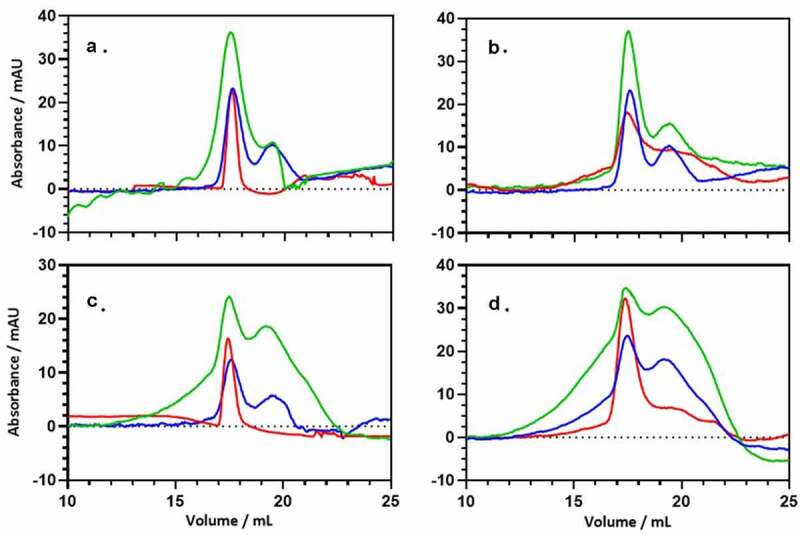
Size-exclusion chromatography (SEC) of PsaA (blue), ANXA2 (red) and the 1:1 mixture (green) of both proteins. (a) SEC was performed with PsaA, ANXA2 (HEK293 T/17), and a 1:1 mixture after incubating each for 1 hr at 25°C respectively. (b) Data was performed with PsaA, ANXA2 (*E. coli*), and a 1:1 mixture after incubating each for 1 hr at 25°C respectively. (c, d) Same proteins as (A) and (B), but incubation occurred for 1 hr at 37°C. The long leading edge in panels (c) and (d) likely originates from fast exchanging, interacting species following incubation

To characterize the interaction of PsaA and ANXA2 *in vitro*, we performed analytical size-exclusion chromatography (SEC). SEC can be used for determining the molecular mass of proteins and separating them under native conditions [27]. Since this technique is largely based on protein identification by size, it can be used to analyze protein – protein interactions [28,29]. Proteins upon interacting with each other tend to form large complexes of varying stabilities. Therefore, based on the elution profile of the individual proteins, any elution peaks of higher molecular mass can be easily identified. All three proteins: PsaA, ANXA2 (*E. coli*), and ANXA2 (HEK293 T/17) have similar molecular masses, and the SEC peaks had correspondingly similar elution profiles (Figure 6). This profile did not change when proteins were incubated separately for 1 hr at either 25°C or 37°C. Each isolated protein eluted at a volume consistent with its monomeric molecular weight on the calibrated SEC column.

Mixtures were then prepared for a 1:1 ratio of PsaA and ANXA2 (*E. coli*) or ANXA2 (HEK293 T/17). After incubation at 25°C for 1 hr, the elution profile at 280 nm for the protein mixture of PsaA and ANXA2 (*E. coli*) as well as PsaA and AXA2 (HEK293 T/17), is largely the sum of the elution profile for the individual, isolated proteins (Figure 6(a, b)). No significant evidence is seen for new, higher molecular weight species eluting at earlier times/volumes. This changes significantly when the proteins were incubated for 1 hr at 37°C, where a significant leading edge is now observed (Figure 6(c, d)). When association is strong and the dissociation rate is slow, an additional, well-defined elution peak at early elution volume is expected. This peak would correspond to the protein–protein complex [30]. However, if the complex undergoes exchange between the free and bound form during the course of the experiment (30 minutes), a broad shoulder is expected as seen here. Under the appropriate circumstances, this shoulder can be interpreted quantitatively to determine an exchange rate [31,32] and analytical ultracentrifugation (AUC) would be more appropriate for determining thermodynamic parameters [33]. Here, we simply demonstrate that the proteins shift to higher molecular weight species upon incubation with heating.

To assess possible structural changes upon binding, we performed circular dichroism (CD) spectroscopy on the proteins and their mixtures. CD is typically used to determine the secondary structure of proteins and any change in secondary structure can be observed for proteins that interact with small molecules such as peptides or nanoparticles [31,34,35]. Secondary structure profiles for both PsaA and ANXA2 looked similar, indicating a high proportion of alpha helices, and this aligns well with their crystal structures (Figure S2(A,B)) [36,37]. To investigate the secondary structural changes in the protein complex, we mixed the proteins in a 1:1 ratio and incubated for 1 hr at either 25°C or 37°C. The CD spectrum of this mixture exhibited only very minor changes from the summed spectra of the individual proteins Figure S2, green vs. purple curves). Significant changes in the CD spectra are expected if one of the proteins is partially folded or becomes unfolded, but that does not appear to occur. Nevertheless, the CD spectra observed are not consistent with unfolded or disordered mixtures of proteins; therefore, the SEC data (Figure 6) most likely does not originate from an anomalous, soluble aggregate. Instead, the interaction observed in SEC more likely represents a fast-dissociating stable complex where the secondary structures of PsaA and ANXA2 are largely retained.

## Binding of ANXA2 to PsaA

**Figure 7.**
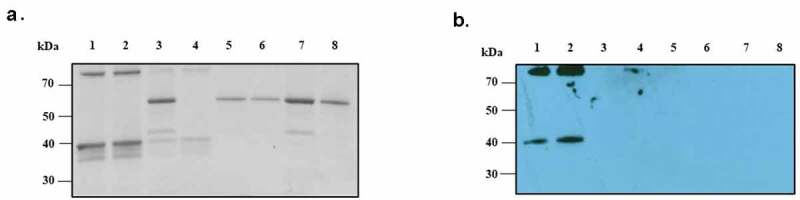
Direct binding interaction between recombinant ANXA2 and PsaA. The bound recombinant ANXA2 or BSA to protein-bound beads or no proteins-beads were separated on SDS-PAGE gel and stained with Coomassie blue G-250 (a) and replicated gel was transferred to PVDF membrane and probed with anti-ANXA2 antibody (C-10) by western blot (b). Bound ANX2 to PsaA-beads (lane 1), bound ANXA2 to PsaA (Mn)-beads (lane 2), bound ANXA2 to BSA-beads (lane 3), bound ANXA2 to no proteins-beads (lane 4), bound BSA to PsaA-beads (lane 5), bound BSA to PsaA (Mn)-beads (lane 6), bound BSA to BSA-beads (lane 7), and bound BSA to no proteins-beads (lane 8)

We investigated the direct binding interaction between recombinant ANXA2 and PsaA proteins. The N-terminus of recombinant PsaA was covalently bound to aldehyde groups of agarose beads and reduced by sodium cyanoborohydride which form stable secondary amine bonds. PsaA-loaded beads were incubated with either recombinant ANXA2 or BSA (control) and following washes, interactions determined by SDS-PAGE gel Coomassie staining and western blot. Recombinant ANXA2 could bind minimally to both BSA-beads and no proteins-beads, (Figure 7, lanes 3 and 4), which indicates incomplete blocking of beads or that washing was not completely effective (Figure 7a). However, stained SDS-PAGE gel and western blot clearly indicate that binding of ANXA2 to PsaA beads was significantly greater than control beads. Similar amounts of BSA bound all four different beads and indicated that binding of ANXA2 to PsaA was specific (Figure 7).

## Discussion

*Streptococcus pneumoniae* is a common colonizer of the nasopharynx and can cause respiratory infection and invasive diseases such as bacteremia, meningitis primarily in children younger than 3 and the elderly [38]. Additionally, the mortality of children under the age of five is higher in developing countries. Since the current pneumococcal vaccines (PCV13, PPSV23) have been licensed, pneumococcal invasive disease has been effectively reduced and a previous case study showed that these vaccines have an effectiveness of 96% in healthy children [39]. However, the most recent vaccine, PPSV23, can only provide protection against 23 of approximately 100 serotypes. Also, several studies indicate that previously common pneumococcal serotypes have been replaced by nonvaccine serotypes [5,10]. Consequently, many groups are studying protein-based vaccines that could provide universal immune response to all different pneumococcal serotypes. The candidate antigens for protein-based vaccine should be genetically conserved to provide broad coverage over different serotypes [40]. Also, protein antigen candidates should be expressed during pathogenesis and reduce colonization of pneumococcus below levels that can result in pneumococcal disease [41]. Thus, selection of target proteins for protein-based vaccines is essential and such targets should ideally prevent both invasive and noninvasive pneumococcal colonization and diseases. There are currently numerous candidates for a protein-based pneumococcal vaccine, including proteins of the histidine triad protein family, PhtD and PhtE, which are surface exposed proteins and expressed in most of pneumococcal strains. A previous study indicated that these proteins could elicit CD4 T cell memory responses against colonization in mice [42]. The choline binding surface protein, PcpA, was also shown to elicit serum antibody response during pneumococcal lung infection and colonization [43]. One of the most important virulence factors of pneumococcus, pneumolysin, is also an attractive candidate for eliciting host immunity and detoxified pneumolysin produces protective, pneumolysin-neutralizing antibodies [44].

Pneumococcal colonization is prerequisite to the develop the pneumococcal infection. The human nasopharynx is the primary colonization site for pneumococcus and multiple proteins are involved in colonization and adherence to host cells. These include PhtD, choline-binding protein A (CbpA or PspC), surface-exposed lipoproteins foldase protein (PrsA), adhesion, and virulence protein A (PavA) and PsaA [12,45]. Previous studies indicated interaction between host cellular receptors and some of pneumococcal proteins that are known to be involved in colonization: polymeric immunoglobulin receptor and laminin receptor for CbpA and fibronectin for PavA [45–47].

Although pneumococcal adhesin proteins have been well characterized, relatively little is known regarding their host cellular receptors. Thus, our group has focused on identification of host cellular receptors for pneumococcal adhesin proteins. PsaA is known as an ABC transporter for manganese. Gram-positive bacteria utilize numerous ABC transporters and lipoproteins to uptake metal ions and other metabolites from the environment. These transporters are also often involved in adhesion and pathogenesis. Previous work by our group and others demonstrated that proteins involved in zinc homeostasis can impact colonization and biofilm formation [34,48–50]. Given its role in Mn^2+^ acquisition, as well as involvement in adhesion, PsaA has also been identified as an important vaccine target.

To identify host cellular receptors for PsaA, we performed far-western blot analysis and identified ANXA2 as a candidate receptor of PsaA. Lentiviral expression of ANXA2 in HEK293 T/17 cells significantly increased adherence of T4R strain and recombinant PsaA or ANXA2 inhibited adherence of T4R strain. These results clearly demonstrated that ANXA2 is a receptor for PsaA. AnnexinA2 is a member of calcium-dependent phospholipid-binding proteins that play a role in multiple cellular processes such as exocytosis, endocytosis, membrane organization, and ion channel conductance [51]. ANXA2 has been shown to be involved in efficient bacterial invasion of epithelial cells by pathogens such as *Rickettisa, Mycoplasma pneumoniae*, and *Pseudomonas aeruginosa* [52,53]. In addition to our findings, another group also demonstrated that pneumococcal surface protein K (PspK) interacts with ANXA2 and promotes adherence to lung epithelial cells [54]. Furthermore, a previous study demonstrated that PsaA also interacts with E-cadherin expressed on nasopharyngeal epithelial cells [18]. While our 2-D far-western analysis did not identify E-cadherin as interacting with PsaA, a faint band of approximately 120kDa was seen in our 1D far-western blot (Figure 1), which may correspond to E-cadherin. These results demonstrated a single pathogen adhesion protein can interact with multiple host receptors and vice versa. To our surprise, the adherence of a PsaA-null mutant to nasopharyngeal epithelial cells was significantly increased compared to wild-type T4R. We are currently investigating why loss of PsaA would increase pneumococcal adhesion to the epithelial surface. A potential explanation is that PsaA could mask other pneumococcal adhesins as was shown for surface protein G (SasG) of *S. aureus* masking the binding of *S. aureus* clumping factor B (ClfB) to cytokeratin of host cell [55]. Additionally, we have previously shown that loss of proteins involved in metal acquisition can impact expression of numerous pneumococcal surface proteins including known and predicted adhesins [34]

Given the high expression of ANXA2 in bronchial epithelial cells and interaction with multiple bacterial adhesion proteins, ANXA2 might be the common receptor exploited by respiratory pathogens to establish colonization. We will continue to investigate the role of ANXA2 in pneumococcal pathogenesis in vivo. This result can imply that ANXA2 can be a common receptor for multiple pneumococcal proteins.ࢭ

## Supplementary Material

Supplemental Material
