# Correction

**DOI:** 10.1080/21505594.2023.2223853

**Published:** 2023-06-13

**Article title**: Pneumococcal surface adhesion A protein (PsaA) interacts with human Annexin A2 on airway epithelial cells

**Authors**: Yoonsung Hu, Nogi Park, Keun Seok Seo, Joo Youn Park, Radha P. Somarathne, Alicia K. Olivier, Nicholas C. Fitzkee, and Justin A. Thornton

**Journal**: *Virulence*

**DOI**: https://doi.org/10.1080/21505594.2021.1947176

It has been noted by the corresponding author that, two undergraduates significantly contributed to the research by helping to construct knockout strains which were included in the final publication. They were mistakenly left off as authors and as both have continued in professional programs, their inclusion on this published work is important to their future careers. An updated author list has been placed below:

Yoonsung Hu^a^, Nogi Park^b^, Keun Seok Seo^b^, Joo Youn Park^b^, Radha P. Somarathne^c^, Natalene Vonkchalee^a^,

Katelyn Jackson^a^, Alicia K. Olivier^d^, Nicholas C. Fitzkee^c^, and Justin A. Thornton^a^

^a^Department of Biological Sciences, Mississippi State University, Mississippi State, USA; ^b^Department of Basic Sciences, College of Veterinary Medicine, Mississippi State University, Mississippi State, USA; ^c^Department of Chemistry, Mississippi State University, Mississippi State, USA; ^d^Department of Population and Pathobiology, College of Veterinary Medicine, Mississippi State University, MS, USA

