# Supplementary figures and images for: Pneumococcal surface adhesion A protein (PsaA) interacts with human Annexin A2 on airway epithelial cells
Source: Virulence. 2021 Jul 8;12(1):1841–54. doi: 10.1080/21505594.2021.1947176 (PMC8274441; doi:10.1080/21505594.2021.1947176)

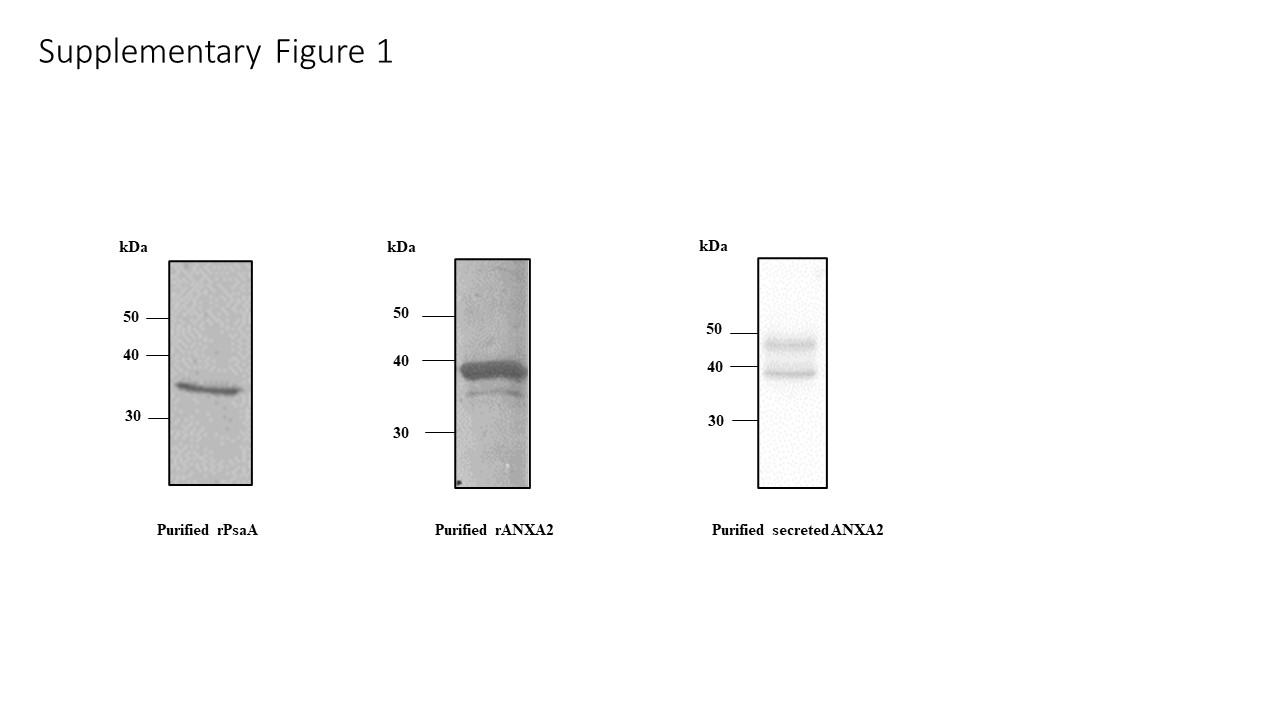

Supplement: Supplemental Material [file KVIR_A_1947176_SM6049.zip › supplementary/Sup Fig 1.JPG]

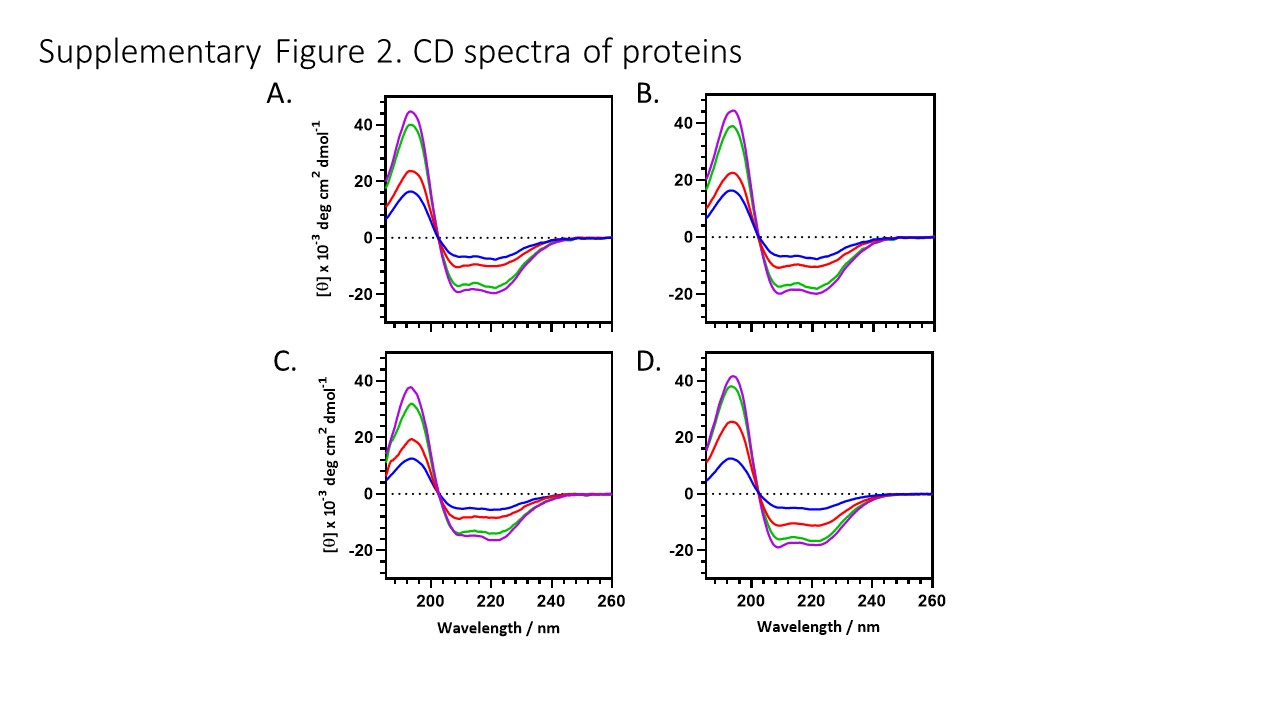

Supplement: Supplemental Material [file KVIR_A_1947176_SM6049.zip › supplementary/Sup Fig 2.JPG]
